# A Lay Journal on Medical Education

**Published:** 1915-12-04

**Authors:** 


### The Hospital

The Workers' Newspaper of Administrative Medicine and Institutional Life, Administration, National Insurance and Health.

No. 1538, Vol. LIX. SATURDAY, DECEMBER 4, 1915. PRICE ONE PENNY.

### A LAY JOURNAL ON MEDICAL EDUCATION.

Our lay contemporary the *Nation* has been led to consider the effect of the war on the medical profession, and to suggest methods for meeting both the immediate difficulties and also those which are likely to appear in the future. The losses due to casualties among doctors on active service, and the diminution in the numbers of medical students are accepted as facts which must necessarily mean an inadequate supply of practitioners and a consequent injury alike to the profession and the nation. Such a position, it is urged, will probably compel the General Medical Council to initiate emergency measures to meet the acuteness of the situation, and the occasion is proposed as a suitable one for reconsidering the whole scheme of medical education. While welcoming the sympathetic tone and good intentions of our contemporary's article, we cannot profess to agree with the proposition just stated. Legislation to meet an emergency may claim a freedom and excuse which would be quite invalid as justification for a permanent policy, and the haste imposed by special circumstances is not the most suitable atmosphere in which to contemplate a far-seeing plan. In an endeavour to compass two ends at one and the same time it is readily possible to fail to attain either, and a scheme for the moment must needs be something different from a durable arrangement; indeed, it would not be unfair to say that the two can hardly be mutually reconciled. Whilst, therefore, we are more than ready to consider any proposition which is likely to help the immediate situation, it seems to us that the mere experience of doing so, not less than the circumstances of the hour, makes the time a very unpropitious one for raising the large question of the ordering of the medical curriculum. An attempt to find one and the same solution for two different problems can hardly anticipate success, and we cannot accept our contemporary's article as a contradiction of this proposition. Moreover, to be quite frank, we must confess to great difficulty in determining among the writer's suggestions those which he would apply to the claims of the moment and those which, on the contrary, are to be part of an abiding reform.

Apart from any technical details of medical education the article in question proposes that the age at which the student commences the curriculum should be raised by "a year or so," and he should be required to possess a degree in Arts or to have attained "a sufficiently good standard of general education." Now, whatever excellencies these proposals may boast, they most certainly will not lead to an early increase of either medical students or medical practitioners; indeed, their effect will be quite in the opposite direction. A lad of sixteen who has passed the preliminary examination may under the present regulations enter a medical school and be registered as a medical student. Youths of this age are too young for military service, and it is to them that we must mainly look in the meantime for new recruits for the profession. If they are forbidden to enter the curriculum for "a year or two," their appearance in the active ranks of the profession will be correspondingly delayed. On the other hand, to ask for candidates among lads of, say, eighteen years is to appeal to those who are just now under extremely strong pressure to take service in the Army. We may hope to get students of sixteen years, but any considerable number from among those who have reached eighteen is in the highest degree improbable. Hence a proposal to increase the age of entrance on the medical curriculum, whatever be its merits in ordinary circumstances, cannot be welcomed amidst the present events, because its adoption, so far from increasing the number of practitioners in the immediate future, would inevitably lessen the supply. Manifestly the same conclusion would follow the demand for a degree in Arts as a necessary preliminary to medical study, and though the writer proposes as an alternative "a good standard of general education," he gives no hint how this is to be tested apart from examination. Such a condition is supposed to exist in the present preliminary examination. It is open to discussion whether this should or should not be modified, but no modification which can be contemplated is likely to increase the number of medical students, and the prescription of an Arts degree is well calculated to diminish the supply. Another proposal is that physics, chemistry, and biology should be studied at school and the examination in them be passed prior to the medical curriculum, but here again there would be no essential alteration in the position relative to the present emergency. Whether the student spent an extra year at school and four years in the medical curriculum or passed all his term as a medical student it would still be five years before he could possibly gain his diploma and be legally qualified to enter on practice. A more plausible suggestion is that the General Medical Council should " waive its first examination," but we very much doubt whether a student untrained in chemistry, physics, or biology would as a result successfully achieve his later studies in some time less than the five years' curriculum, and he might, indeed, find his final success considerably delayed.

We repeat that we are far from resenting proposals from the outside, but we are driven to the conclusion that the writer in the *Nation* has mixed together two questions which are essentially distinct and that confusion of thought has been the result. There may, too, arise a doubt whether he is personally well equipped for the task he has undertaken, seeing that he describes the medical student as not sufficiently " serious," and is under the impression that all the ordinary practitioner remembers of chemistry is that when " this liquid poured into that tube produces a blue colour " the disease is diabetes! His general propositions do not much invite us, but the ludicrous blunder of his technical illustration must excite general commiseration. For him, most certainly, we cannot " waive " the first examination.

